# Patterns in Abundance, Cell Size and Pigment Content of Aerobic Anoxygenic Phototrophic Bacteria along Environmental Gradients in Northern Lakes

**DOI:** 10.1371/journal.pone.0124035

**Published:** 2015-04-30

**Authors:** Lisa Fauteux, Matthew T. Cottrell, David L. Kirchman, Carles M. Borrego, Maria Carolina Garcia-Chaves, Paul A. del Giorgio

**Affiliations:** 1 Groupe de Recherche Interuniversitaire en Limnologie (GRIL), Département des sciences biologiques, Université du Québec à Montréal, CP 8888, Montréal, Québec, Canada; 2 School of Marine Science and Policy, University of Delaware, 700 Pilottown Rd., Lewes, DE 19958, United States of America; 3 Group of Molecular Microbial Ecology, Institute of Aquatic Ecology, University of Girona, Campus de Montilivi, E-17071, Girona, Spain; Argonne National Laboratory, UNITED STATES

## Abstract

There is now evidence that aerobic anoxygenic phototrophic (AAP) bacteria are widespread across aquatic systems, yet the factors that determine their abundance and activity are still not well understood, particularly in freshwaters. Here we describe the patterns in AAP abundance, cell size and pigment content across wide environmental gradients in 43 temperate and boreal lakes of Québec. AAP bacterial abundance varied from 1.51 to 5.49 x 10^5^ cells mL^-1^, representing <1 to 37% of total bacterial abundance. AAP bacteria were present year-round, including the ice-cover period, but their abundance relative to total bacterial abundance was significantly lower in winter than in summer (2.6% and 7.7%, respectively). AAP bacterial cells were on average two-fold larger than the average bacterial cell size, thus AAP cells made a greater relative contribution to biomass than to abundance. Bacteriochlorophyll *a* (BChl*a*) concentration varied widely across lakes, and was not related to AAP bacterial abundance, suggesting a large intrinsic variability in the cellular pigment content. Absolute and relative AAP bacterial abundance increased with dissolved organic carbon (DOC), whereas cell-specific BChl*a* content was negatively related to chlorophyll *a* (Chl*a*). As a result, both the contribution of AAP bacteria to total prokaryotic abundance, and the cell-specific BChl*a* pigment content were positively correlated with the DOC:Chl*a* ratio, both peaking in highly colored, low-chlorophyll lakes. Our results suggest that photoheterotrophy might represent a significant ecological advantage in highly colored, low-chlorophyll lakes, where DOC pool is chemically and structurally more complex.

## Introduction

Aerobic anoxygenic phototrophic bacteria are members of Proteobacteria that synthesize bacteriochlorophyll *a* (BChl*a*), which is incorporated into a functional photosynthetic system and allows them to carry out anoxygenic photosynthesis only under aerobic conditions. The photo-induced cyclic electron transport results in the production of energy, but AAP bacteria do not possess the key enzymes needed for CO_2_ fixation in the Calvin cycle, thus they are incapable of growing without a source of organic carbon [1]. Light therefore provides an energy supplement to their mainly chemotrophic metabolism, allowing them to function as photoheterotrophs [2,3]. Accumulated evidence suggests that AAP bacteria, as a group, are a diverse, dynamic and likely globally significant component of aquatic microbial communities [4–6].

AAP bacteria inhabit a wide range of aquatic systems; their abundance and distribution have been explored in open-ocean [7–11], coastal and estuarine habitats [12–14] as well as in lakes [15–18] and rivers [19]. The contribution of AAP bacteria to total bacterial abundance has been shown to be in the range of 1 to 11% in marine systems [9,12,20] and to vary considerably more, between 1 to 30%, across freshwater ecosystems [15,22]. Factors such as association to particles [14], temperature [13,18,22], light availability [12,18,23], phosphorous [16], chlorophyll *a* (Chl*a*) [20,21] and dissolved organic carbon (DOC) [15,18] have been identified as the environmental drivers that best explain this variation, but the role of these drivers is still not well understood. AAP abundance has been shown to increase with Chl*a*, total phosphorous (TP) content or with DOC concentration in a handful of marine and freshwater surveys [16,18,20,21,24]. The positive trend observed between AAP abundance and trophic status [16,20] does not support the hypothesis, initially held by some studies [15,25], that the ability of these bacteria to use light should be especially advantageous in nutrient-poor environments. Moreover, the negative relationship between AAP abundance and water transparency observed in some marine and freshwater studies [18,23] and the lack of a light enhancement on AAP activity in short incubations in artic and estuarine waters experiments [6,26] seems to be in conflict with the potential ecological advantage that photoheterotrophy might confer to this group. AAP bacteria are likely reacting to several superimposed gradients, and are probably regulated by multiple factors, thus the question regarding the environmental conditions that explain AAP abundance and performance remains to be solved.

The greater heterogeneity of freshwater systems relative to marine environments offers an opportunity to address the role of most of the identified drivers of AAP abundance and distribution, but particularly to study the effects of the overlapping gradients of DOC, nutrients and light on this group of photoheterotrophic bacteria. In lakes, the composition of DOC varies in quantity and quality depending on the balance between local primary production and the input of organic compounds from terrestrial sources [27,28]. Lakes exhibit a large gradient of DOC that differentially combines a highly labile pool of low molecular weight compounds of algal and terrestrial origin with a more refractory pool of heavy and highly coloured substances mostly of terrestrial origin [29]. Interestingly, this highly coloured pool of DOC has an impact on the aquatic light climate [30], which in turn shapes the environmental conditions for the photoheterotrophic organisms. Besides this spatial DOC heterogeneity, lakes in cold temperate regions develop ice and snow cover in winter, a condition that further modifies the availability of light and results in another ecologically interesting scenario for the regulation of AAP bacteria.

Here we present results of a large-scale study aimed at assessing the distribution of AAP bacteria across a wide range of temperate and boreal lakes in northern Québec. We explored the spatial and temporal patterns in the abundance, cell size and pigment content of AAP bacteria, along with the environmental factors that influence these patterns. The lakes were chosen to maximize both trophic (in terms of phosphorous and chlorophyll *a*) and dissolved organic carbon (DOC) gradients. We further assessed the presence of AAP bacteria in winter under ice-cover and along depth profiles in stratified lakes, to explore the possible interaction between resources and light availability.

## Materials and Methods

### Sites and sampling

This study targeted lakes in three distinct regions of northern Québec: The Eastmain River region of boreal Québec (52°14’N, 75°W), which contains an extensive freshwater network covering over 20% of the landscape. This region is characterized by mature evergreen forest dominated by black spruce (*Picea mariana*). The Laurentian region is located in the Canadian Shield, north of Montréal (45°59’N, 74°01’W), dominated by granitic bedrock and mostly covered by mixed forest (>95%). The Eastern-Townships region is located east of Montréal (45°24’N, 72°12’W) in the St-Lawrence Lowlands, dominated by sedimentary geology that results in a higher average pH and alkalinity lakes [31].

In all, 43 lakes were sampled, 17 in the boreal region (S1 Table), 13 in the Eastern Townships (S2 Table), and 13 in the Laurentians (S3 Table). Of these, 26 were sampled only once, whereas 17 were visited on more than one occasion. Thirteen out of these 17 lakes were sampled three to six times, including at least one under-ice sample. No specific permissions were required for sampling the lakes, all of which have public access. The field studies did not involve endangered or protected species, or vertebrates.

Sampling was always conducted over the deepest part of the lakes and at each sampling location, we carried out a complete vertical profile of temperature, oxygen, and conductivity, using a 600 XLM-M probe (YSI) and we measured Secchi disk depth. The water samples were only collected from fully aerobic layers, always at one-meter depth for surface samples and additionally deeper when metalimnia were sampled, using a peristaltic pump. During the ice-cover period, sampling was carried out through a hole made through the ice. In all cases, the samples were collected into 18-L carboys and processed in the lab within 4 to 6 hours.

### Abundance and size of AAP bacteria

Samples for the microscopic enumeration of total and AAP bacterial abundance were fixed immediately after collection with 1% glutaraldehyde final concentration and kept refrigerated in the dark. Back in the lab, samples were filtered onto 0.2 μm pore-size black polycarbonate filters (110656, Whatman) and immediately stored at -80°C. Total prokaryotic and AAP bacterial abundance and average cell size were determined by infrared epifluorescence microscopy, as described by Cottrell *et al*.[32]. In brief, filters were stained for five minutes with 1μg ml^-1^ 4,6-diamidino-2-phenylindole (DAPI) in 2X phosphate-buffered saline solution. AAP and total bacteria were counted using an Olympus Provis AX70 microscope, fitted with a charge-coupled-device camera capable of infrared detection (Intensified Retiga Extended Blue Qimaging) and image analysis software (ImagePro Plus, Media Cybernetics). To enumerate total bacteria (DAPI-stained cells) and to discriminate and count AAP bacteria, twenty fields of view were assessed per slide, and four images were recorded for each field of view, each at a different excitation and emission wavelength. The excitation and emission wavelengths were as follows: 360 nm and 460 nm for DAPI-stained cells; 390 and 750 nm for AAP bacteria; 480 and 660 nm for cells containing Chl*a*, and 545 and 610 nm for cells containing PE. In addition to abundance, cell size was also determined by image analysis from the DAPI-stained cells using the integration method proposed by Sieracki [33].

For a subset of 13 samples, we prepared replicate filters to compare the variation in average counts between two fields from the same filter with the variation between fields from the two replicate filters. The average coefficient of variation between fields within a sample was similar to the average for fields from replicate filters (33.3 ± 8.4% and 28.9 ± 3.6%, respectively).

### Pigment analysis

Water samples (from 1 to 18L, depending on the lake and sample type) were filtered through 47mm-diameter GF-F glass microfiber filters (Whatman) and stored at -80°C until processing. This filter has a nominal pore size of 0.7μm, but our previous experience has shown that it effectively retains smaller particles, especially when large volumes of water are passed and the filter tends to clog. All further manipulations were carried out at room temperature and under dim light to prevent photo-oxidation of labile pigments. Filters were thawed and cut into small pieces using sterile scissors. Pigments were extracted in acetone:methanol (7:2 vol:vol, Scharlau HPLC Grade) after a mild sonication for 30s at 4°C using a B-Braun Labsonic 2000 disruptor. Extracts were stored overnight at -30°C, then clarified by vacuum filtration. Eluents from this first extraction were collected in light-protected sterile Falcon tubes and the remaining filter fiber matrix was re-used for two additional extraction steps to ensure complete pigment recovery. Sample eluents were then combined and stored at -30°C until analysis by reversed-phase HPLC, according to Borrego and Garcia-Gil [34]. This method takes longer (60 min) than other chromatographic programs commonly used to detect BChl*a* from AAP bacteria [35][36], but it offers a better resolution on the separation of other algal and bacterial pigments that can interfere in the identification of BChl*a* (*e*.*g*. bacteriochlorophylls and carotenoids from anoxygenic photosynthetic bacteria that can be present in deep, anoxic water layers of some lakes). BChl*a*, Chl*a* and chlorophyll *b* (Chl*b*) peaks were identified from their retention times and absorption spectra using the online diode array detector, coupled to the chromatographic system. Peak areas were recorded at 771nm (BChl*a*), 432nm (Chl*a*) and 465nm (Chl*b*) and converted to concentrations after calibration using pigment standards and appropriate extinction coefficients [37].

### Chemical analysis

Total phosphorus (TP) was measured using the molybdenum-blue method, after potassium persulfate digestion. DOC was measured on a TOC1010 total carbon analyzer (OI Analytical) using a high-temperature sodium persulfate oxidation method. For Chl*a* determinations, we filtered one liter of water on 47mm-diameter GF-F glass microfiber filters (Whatman), extracted the filter in hot ethanol, stored it overnight at 4°C, then determined the absorbance of the extract at 665nm and 750nm, using a UV/Vis UltroSpec 2100 spectrophotometer (Biochrom Ltd.). We have used this estimate for all subsequent analyses, but note that there was good agreement between the spectrophotometric and the HPLC-based chlorophyll estimate described in the previous section.

### Statistical Analysis

We used least squares linear regression to assess links between response and explanatory variables, while stepwise multiple regression was used to identify combined effects. The data were log-transformed to assure normality and homoscedasticity of model residuals, which was confirmed using a Shapiro-Wilk test. ANOVA was used to test seasonal differences in the key variables. For all the statistical analysis we used the software JMP 9.0 (SAS Institute Inc., Cary, NC, USA).

## Results

Our measurements include both seasonal sampling from several lakes, as well as single-point summer sampling for a wider range of lakes (see S1–S3 Tables for detailed information). We used only the summer (June to September) data to assess large-scale spatial patterns across lakes; these data span a broad range of environmental gradients. Chl*a* and TP concentrations varied 100-fold among those lakes (0.8 to 55.4 μg L^-1^, and 3.6 to 177 μg L^-1^, respectively), whereas DOC varied 6-fold (2.3 to 12.8 mg L^-1^).

Even though all the lakes sampled were thermally stratified in summer and winter, the vertical profiles of dissolved oxygen confirmed the fully aerobic conditions of 41 out of the 43 lakes sampled. Croche and Bran-de Scie were the only two lakes with a well-developed anaerobic hypolimnion. These two lakes typically had high thermal stability during stratification (Schmidt stability index of 72 and 30 J m^-2^ respectively) [38], which prevented any significant mixing of aerobic and anaerobic layers, reducing the possibility of having anaerobic prokaryotes in the oxygenated epilimnetic and metalimnetic layers. We can therefore safely assume that the microscopic enumeration of bacteria and the extracted BChl*a* originate from AAP bacteria.

### Magnitude of AAP bacterial abundance, cell size and total biovolume

**Fig 1:**
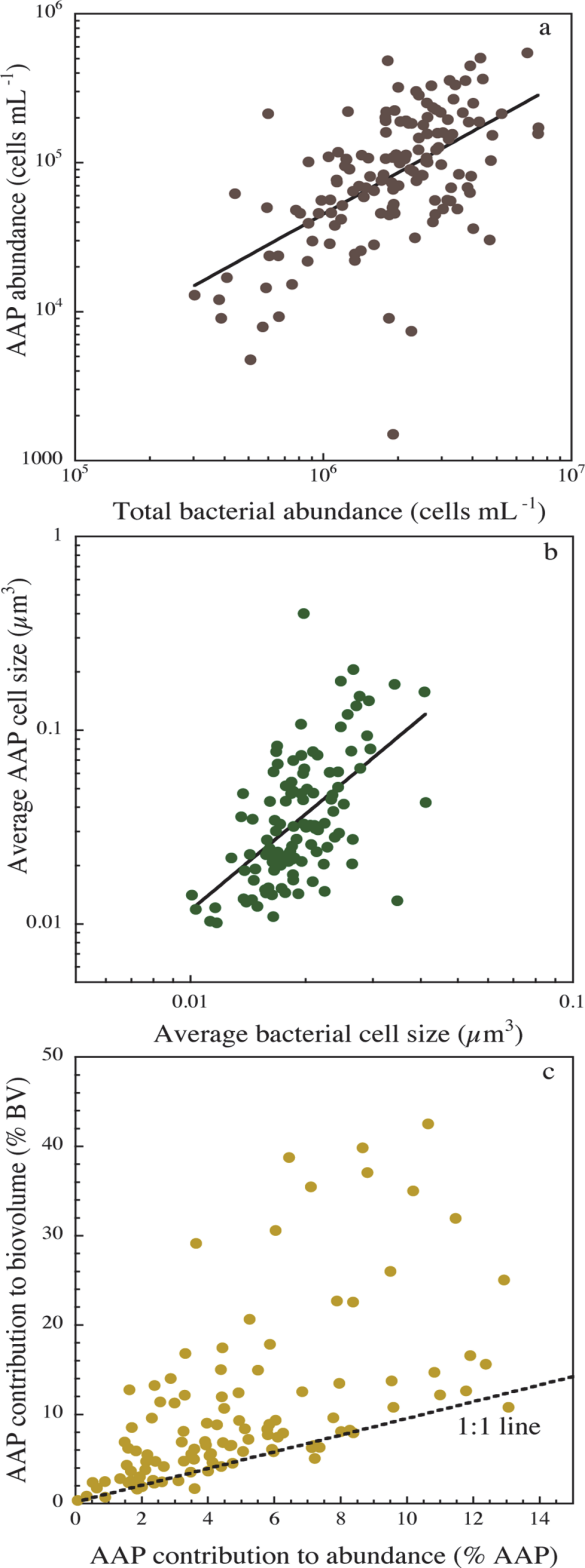
Relationship between total prokaryote abundance and (a) AAP bacterial abundance and (b) cell size. **(c) Contribution of AAP bacteria to total abundance (%AAP) versus their contribution to total bacterial biovolume (%BV).** The line in (a) and (b) is the least-square regression, and details of the regression models are given in Table 1. The line in (c) is the 1:1 line. Data have been log-transformed, and the plots comprise all data collected between July 2007 and October 2008.

AAP bacteria were detected in all the 140 samples (see S4 Table for detailed abundance data) and their abundance spanned two orders of magnitude, from 1.51x10^3^ to 5.49x10^5^ cells mL^-1^ (mean 1.21x10^5^ ± 9.1x10^3^ cells mL^-1^); the resulting proportion of AAP bacteria varied from 0.07% to 37.3% (mean 5.40 ± 4%). AAP bacterial abundance increased with total prokaryotic abundance (Fig 1A), but the relative proportion of AAP bacteria was not correlated with total prokaryotic abundance. Total bacterial cell size varied four-fold across all samples, from 0.01 to 0.04 μm^3^ (mean 0.02 ± 0.0005 μm^3^), whereas AAP bacterial cell size varied twenty-fold, from 0.01 to 0.21 μm^3^ (mean 0.04 ± 0.004 μm^3^), and both were positively related (Fig 1B). The log-slope of this relationship was 1.64, which suggests that AAP bacterial cell size is considerably more variable than the average bacterial size over the range of lakes. Moreover, AAP bacterial cells were on average two-fold larger than average bacterial cells (Student *t*-test *n* = 140, p < 0.0001). The total bacterial biovolume, calculated as the product of cell size and abundance, varied 30-fold (5 to 178 x 10^3^ μm^3^ mL^-1^), whereas the total biovolume of AAP bacteria varied 10-fold (0.2 to 22 x 10^3^ μm^3^ mL^-1^). Since AAP bacterial cells where significantly larger, their average contribution to total bacterial biovolume was significantly higher (mean 9.5% ± 0.8) than their average contribution to total abundance (Student *t*-test, n = 140, *p* < 0.0001) (Fig 1C).

### Bacteriochlorophyll *a* concentrations

BChl*a* concentrations varied from 11 to 47 ng L^-1^ in the surface waters of the lakes sampled over the open water season, and from 5 to 47 ng L^-1^ along depth profiles. Contrary to previous results [25], there was no relationship between AAP abundance, determined using IR microscopy, and the concentration of BChl*a*, measured by HPLC. There was a four-fold range in BChl*a* concentration for any given AAP cell abundance (S1 Fig).

### Temporal variability

**Fig 2:**
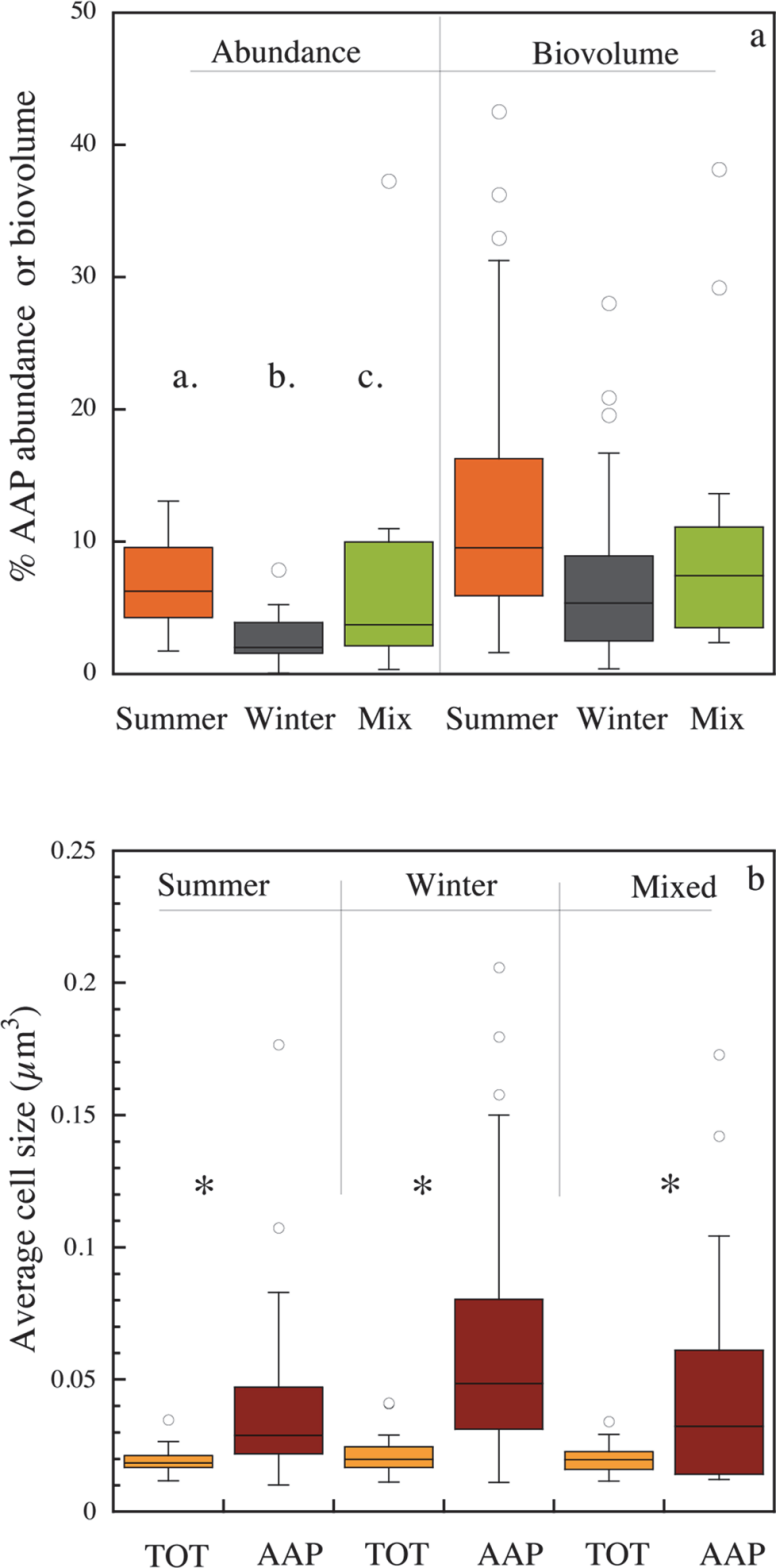
Seasonal patterns in the contribution of AAP bacteria to (a) total bacterial abundance and to biovolume, and (b) in the average cell size of AAP and total bacteria. The box-and-whisker plots show the average, 75% quartiles and extremes values, and stars indicate significant differences (p<0.05).

There were clear temporal patterns in both abundance and cell size of AAP bacteria over seasonal time scales. The data were grouped into three broad seasonal categories based on time of sampling, including winter (ice cover period), summer (stratification period), and a mixed period, which included samples from spring and autumn. Total prokaryotic abundance varied little across seasons, whereas AAP bacterial abundance clearly declined in winter, and thus there were significant seasonal differences in the contribution of AAP bacteria to total abundance (ANOVA, *n* = 99, *p* < 0.0001; Fig 2A). The relative contribution of AAP bacteria was lowest in winter (mean 2.6 ± 0.3%), highest in summer (mean 7.7 ± 0.8%), and intermediate during spring and autumn mixing (mean 5.2 ± 0.9%). AAP bacterial abundance did not correlate with water temperature, yet the proportion of AAP bacteria significantly increased with water temperature (Table 1). AAP bacteria contributed to total volume more than they contributed to abundance (Fig 2A). The average bacterial cell size remained relatively constant, and was not significantly different among seasons (overall mean 0.02 ± 0.007 μm^3^, Fig 2B), whereas the average AAP bacterial cell size varied significantly among seasons (ANOVA, n = 82, p < 0.005). On average the cell size of AAP bacteria was lowest in winter. Within each period, AAP bacteria were always significantly larger than the average bacteria (Fig 2B). AAP bacterial cell size decreased with increasing water temperature (Table 1).

**Table 1:** Regression models relating AAP bacteria variables to total bacteria or describing the cross-lake patterns in AAP bacteria and total bacteria as a function of environmental variables.

Model	*N*	*r* ^*2*^	*P*
Log AAP = -2.04 + 0.92 log BA	139	0.34	< 0.0001
Log AAP size = 1.3 + 1.64 log total size	113	0.35	< 0.0001
Log AAP BV = -1.7 + 1.11 log total BV	113	0.46	<0.0001
%AAP = 2.1 + 0.23 (Water Temperature)	88	0.32	< 0.0001
AAP size = 0.072–0.0019 (Water Temperature)	84	0.15	0.0004
Log BA = 6.5–0.31 log (Secchi)	38	0.27	0.0009
Log AAP = 5.2 – 0.51 (log Secchi)	38	0.26	0.001
Log BA = 6.3 + 0.23 log (Chl*a*)	38	0.21	0.004
Log AAP = 4.57 + 0.70 log (DOC)	38	0.21	< 0.004
%AAP = 2.67 + 0.5 (DOC)	38	0.22	0.0027
Log AAP BV = 2.74 + 1.0 log (DOC)	29	0.23	0.008
Log %BChl*a* = 0.47 – 1.26 log (Chl*a*)	30	0.84	< 0.0001
Log SpBChl*a* = 0.22 – 0.89 log (Chl*a*)	30	0.5	< 0.0001
Log %AAP = 0.47 + 0.67 log (DOC:Chl*a*)	34	0.39	< 0.0001

AAP: Aerobic anoxygenic phototrophic bacterial abundance; BA: Total bacterial abundance; Total size: Total prokaryotic cell size; BV: total biovolume; SpBChl*a*: Pigment content per cell; %BChl*a*: relative contribution of BChl*a* to total pigments; Secchi: Secchi disc depth; DOC: Dissolved organic carbon.

### Depth-related variability

**Fig 3:**
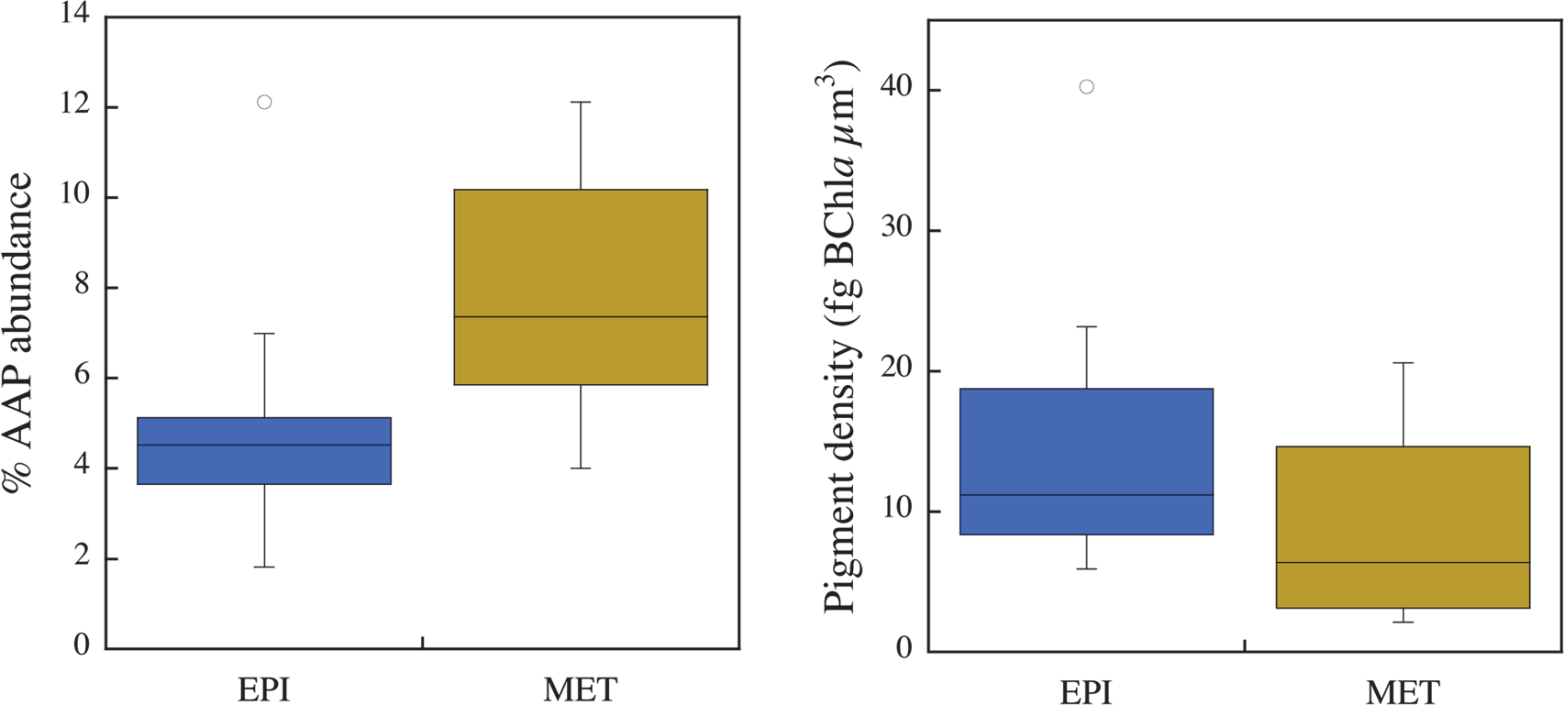
The average and distribution of the relative abundance of AAP bacteria (%AAP), and of the pigment content per cell volume (fg BChl*a* μm^-3^) in the epilimnion and in the metalimion of selected lakes. The box-and-whisker plots show the average, 75% quartiles and extremes values in the epilimnetic and metalimnetic layers during lake summer stratification.

The vertical profiles in four of the temperate lakes (lakes Croche, Connely, Bowker, and Brand-de-Scie), sampled in July 2008 during summer stratification, revealed relatively constant total bacterial abundance at all depths, and increasing AAP bacterial abundance with depth. AAP bacterial abundance was on average highest in the metalimnion; overall, the contribution of AAP bacteria to total bacterial abundance increased with depth (Fig 3). In several vertical profiles we analyzed the abundance of AAP but also BChl*a* concentration (ng L^-1^), the pigment content per cell (fg BChl*a* cell^-1^), and the pigment density per cell (fg BChl*a* μm^-3^). The average BChl*a* concentration (Epi: 25.3 ± 5.9 and Meta: 26.9 ± 2.5 ng BChl*a* L^-1^) and the pigment content per cell (Epi: 0.43 ± 0.1 and Meta: 0.8 ± 0.6 fg BChl*a* cell^-1^) in the metalimnion were not different from the concentration in the epilimnion. However, there was a greater difference between depths in the pigment density per cell (Fig 3). AAP bacterial cells in the epilimnion contained almost two-fold more BChl*a* (14.9 fg BChl*a* μm^-3^) than those in the metalimnion (8.9 fg BChl*a* μm^-3^). Metalimnetic AAP bacteria were on average larger, albeit not significantly (data not shown), and thus some of the differences in pigment density were the result of increases in cell volume without concomitant increases in pigment content.

### Spatial variability among lakes

**Fig 4:**
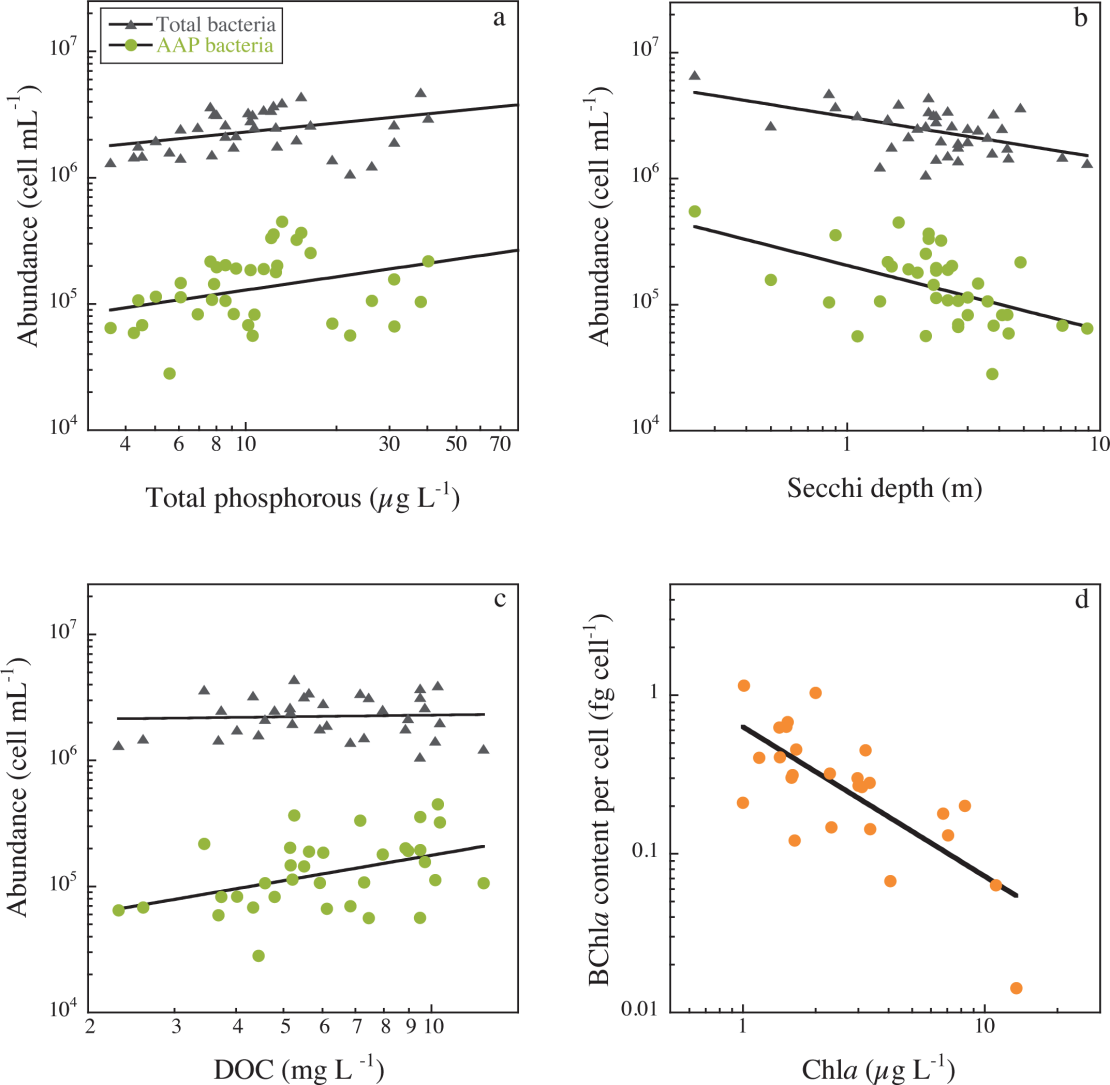
The relationship of total bacterial abundance (triangles) and AAP bacterial abundance (circles) with total phosphorous concentration (a), Secchi depth (b), and DOC concentration (c). **BChl*a* contents per cell as a function of mean lake chlorophyll concentration (d)**. Data have been log-transformed, the line is the least-square regression, and details of the regression models are given in Table 1.

**Fig 5:**
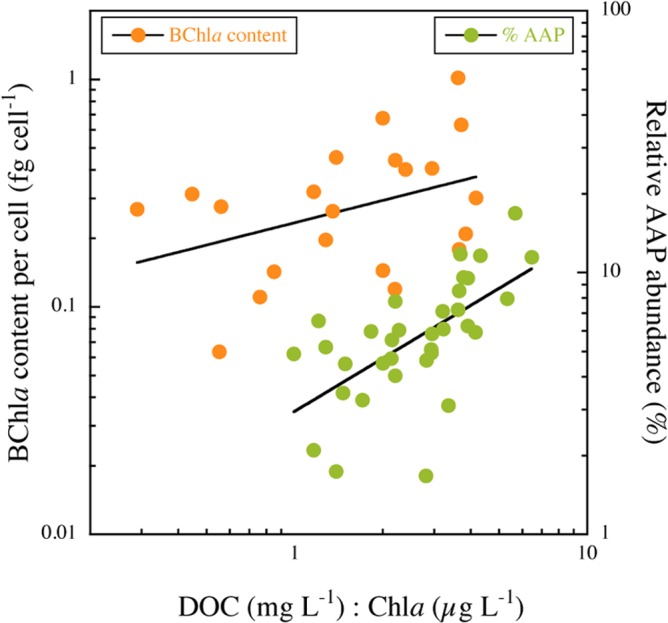
The contribution of AAP bacteria to total bacterial abundance (%AAP, squares) and the BChl*a* content per cell (circles) as function of the DOC to Chl*a* (DOC:Chl*a*) ratio. The data have been log-transformed, the line is the least-square regression, and details of the regression models are given in Table 1.

Since not all lakes had similar temporal coverage, we used the summer data from the 43 lakes to assess patterns in total bacteria and AAP bacteria variables along environmental gradients. Total bacterial and AAP abundance increased with total phosphorous (Fig 4A) and Chl*a* (Table 1), and both were negatively correlated with Secchi disk depth (Fig 4B and Table 1). AAP bacterial abundance was significantly positively related to DOC, whereas total bacterial abundance did not vary with DOC (Fig 4C and Table 1). As a result, the contribution of AAP bacteria to total abundance was positively related to DOC (Table 1). Interestingly, the average size of AAP bacteria also tended to increase with DOC concentration, and consequently AAP bacterial biovolume also tended to significantly increase with DOC (Table 1). BChl*a* concentration was not significantly correlated to Chl*a* and remained relatively constant across the trophic gradient (data not shown). The cell-specific BChl*a* content, however, decreased exponentially across the Chl*a* gradient (Fig 4D and Table 1). The best individual predictor of both relative AAP bacterial abundance and of the cell-specific BChl*a* content was the ratio of DOC to Chl*a* (Fig 5 and Table 1). The highest relative abundance of AAP bacteria and the highest cell-specific BChl*a* contents occurred in lakes having both the highest DOC and lowest Chl*a* concentrations (Table 1).

## Discussion

We detected AAP bacteria in samples from all 43 lakes studied, thus confirming the widespread presence of this group in northern lakes having different biological, chemical and physical features. We found that the relative contribution of AAP bacteria to total prokaryotic abundance ranged from less than 1% to 37%, which is well within the range previously reported for lakes [15,16,18,21]. AAP bacteria clearly tracked total cell abundance, both across systems, and within systems over time. The covariation of AAP and total bacterial abundance is implicit in several previous studies [13,24,39] but has seldom been actually quantified [20][40]. This pattern of covariation would suggest that AAP bacteria respond to the same basic environmental drivers and are subject to a similar overall regulation as the bulk of the bacterial community. However, AAP bacteria do appear to be more dynamic than the community as a whole, which was revealed by a larger spatial and temporal variability in cell abundance and cell size relative to the total bacterial community.

There was significant seasonal variability in both the absolute and the relative abundance of AAP bacteria. A clear seasonal pattern observed in these lakes was a steep decline in absolute and relative abundance of AAP bacteria in winter during ice cover. Few studies have explored the seasonal dynamics of AAP bacteria, especially under the ice, and these are in general agreement with our current results. For example, Mašin *et al*. previously reported seasonal variations in the abundance of AAP bacteria in two European lakes [15]. These authors observed maximum AAP numbers in summer and minimum values in winter, although not necessarily under ice cover. Similarly, AAP bacterial abundance was observed to decrease from summer to winter in coastal waters of the West Antarctic Peninsula [26], in the Northwestern Mediterranean Sea [12], as well as in Arctic waters [41]. Our results together with previous observations indicate that winter conditions are either unfavourable for the growth of AAP bacteria, and/or that AAP bacteria may persist in the system but may regulate their light harvesting pigment content and therefore go undetected by epifluorescence microscopy.

Despite the clear AAP abundance decline observed in winter, AAP cells containing BChl*a* were still detectable under the ice in these northern lakes, as was previously reported for the Arctic Ocean [41]. Recently, Kirchman and Hanson [42] estimated the energy yields and costs associated to photoheterotrophy in AAP bacteria. According with their calculations, light intensities greater than 400 μmol photons m^−2^ s^−1^ are required for phototrophy to yield sufficient energy to meet the maintenance cost of an AAP cell. Therefore, the presence of the photosynthetic apparatus probably offers only a small metabolic advantage under the low light intensities characteristic of ice-covered surface waters. This issue is closely related to the strategies of pigment production and regulation in AAP bacteria. The lack of relationship between AAP abundance and BChl*a* concentration (S1 Fig) implies that the concentration of pigment per cell varies greatly and that a higher number of AAP is not necessarily translated into a higher concentration of total pigment. In this regard, we found that pigment density (as fg BChl*a* μm^-3^) was on average lower in the metalimnion, in agreement with previous studies that have reported a decline in AAP pigment content per cell with depth [9,32]. This pattern could reflect a lower dependence of AAP bacteria on phototrophy as light is attenuated, and the existence of a threshold of light intensity for the expression of phototrophy, although we are still far from understanding the role of phototrophy in the ecology and bioenergetics of AAP bacteria.

Light availability has been proposed as the main driver of the temporal variation in relative AAP bacterial abundance. Shorter days and low light intensity have been previously suggested as drivers of the changes in abundance of this photoheterotrophic group of bacteria during winter [12,26]. The role of light in governing AAP abundance and activity, however, is still unclear [40,43]. Our results together with another previous freshwater study [18] evidenced a negative relationship of AAP abundance and water transparency, which is contrary to the hypothesized positive effect of light. Our results are in agreement with two recent studies that quantified the single cell activity of AAP bacteria (using micro-autoradiography) which reported no light enhancement of AAP growth [6,26]. We might therefore hypothesize that whereas light-derived energy may have little direct effect on growth, it might nevertheless play a role in sustaining other aspects of cell energy metabolism.

We have shown that the AAP bacterial abundance and biomass increased with phosphorous concentration and overall system productivity. In addition, specific BChl*a* content decreased exponentially from oligotrophic to eutrophic conditions, which would suggest that the heterotrophic metabolism of AAP bacteria, which is linked to DOC consumption, is favoured in the most productive systems, as has been previously shown [20]. We also found that the relative contribution of AAP bacteria to total prokaryotic abundance did not reach its maximum in the most oligotrophic lakes, but rather followed a gradient in the DOC:Chl*a* ratio, peaking in high DOC, low chlorophyll lakes. Interestingly, we found that the BChl*a* content per cell clearly followed the same gradient, also peaking in dystrophic lakes. These results suggest that it is in high DOC, low chlorophyll lakes where AAP phototrophy is mostly expressed.

Our results indicate that the highly eutrophic lakes tend to deviate from this general DOC:Chl*a* pattern, suggesting that it may not be the absolute amount of organic C that is relevant to the performance of AAP bacteria, but rather its composition. As the DOC concentration increases and lakes become more dystrophic, the C pool becomes chemically and structurally more complex [29,44]. The presence of a phototrophic pathway may provide an energetic primer that allows AAP bacteria to utilize complex and recalcitrant substrates, for example, humic compounds, which would otherwise be energetically inefficient to access, in a process analogous to cometabolism [45]. In agreement with this hypothesis, Eiler *et al*. and Mašin *et al*. reported that the prevalence of BChl*a* synthesis genes [17] and, the relative abundance of AAP [16] was markedly higher in humic relative to clear-water lakes. Likewise, Ferrera *et al*. [12] observed higher numbers of AAP bacteria and higher pigment content when DOC accumulates annually and becomes chemically and structurally more complex at the coastal NW Mediterranean Sea. Cuperová *et al*. [12] also found that DOC availability was the main factor influencing AAP distribution across alpine lakes, in agreement with Hauruseu and Koblížek [3] who postulated that light-derived energy might play only an auxiliary function by increasing the efficiency of DOC utilization. Interestingly, a previous study on freshwater AAP bacterial diversity [46] has shown that humic acidic conditions and/or the recalcitrant nature of the DOC pool in lakes favour the growth of particular groups of AAP bacteria, mostly related to the genre *Methylobacterium* and *Sphingomonas*. Humic matter enrichment experiments [47] and the genome annotation of a *Sphingomonas* sp. strain [48] further demonstrated that AAP bacteria from these genre participate in humic matter degradation. Therefore, it might be hypothesized that the AAP community composition changes along the observed DOC:Chla gradient, with increasing dominance of AAP taxa that can participate in the degradation of humic compounds as the DOC pool becomes chemically and structurally more complex.

We have further shown that lake AAP bacteria are significantly larger than the average bacterial cells in these communities, as was noted before for marine systems [9,26,33]. One of the consequences of the larger size of AAP bacteria is that their contribution to total bacterial biomass is almost two-fold higher than their contribution to abundance (overall average of 10% and 6%, respectively). Furthermore, cell size provides valuable insight into the trophic significance of AAP bacteria. A larger size renders AAP cells more vulnerable to grazers [49,50]. If AAP bacteria are more intensely grazed than the bulk bacterial community, their production will be then preferentially transferred to higher trophic levels. The only study so far to have explored the top-down control of AAP abundance, showed that AAP bacteria were subjected to high grazing pressure, and this process was responsible for the low densities of this fast-growing group [4]. In addition to being larger than the average bacterial cells, AAP cells appear to be more dynamic in terms of size: we observed that AAP cell size varied twenty-fold across all lake samples whereas total bacterial cell size varied only four-fold; previous studies have also reported large variations in AAP cell size [26,51]. We hypothesize that shifts in top-down pressure on AAP bacteria probably contribute to the variation in their cell size.

Linked to the issue of the potential ecological implications of photoheterotrophy is the question of whether cells that contain BChl*a* and express the associated metabolic function can be considered a true functional group, sharing common regulation and environmental requirements. It is now clear that AAP bacteria are ubiquitous, we know that the *puf* operon is widely distributed within the Proteobacteria [17,18,52], and that many coexisting phylotypes can express this function within a given environment [12,53], but it still is not established to what extent photoheterotrophy plays a role in the distribution of these bacterial taxa. There are two scenarios under which AAP bacteria could form an ecologically coherent group: 1) the metabolic functionality conferred by this trait is of such relevance that it overrides differences in other traits; 2) the functionality conferred by AAP metabolism is not in itself determinant, but its presence is linked to other key functional traits, such as tolerance to environmental conditions, metabolic plasticity, and functional capacities.

Our results indicate that AAP bacteria are far from being a homogeneous group in terms of cell size and pigment content, and that the wide range of variation observed within lakes in time and across lakes, may be a reflection of the potentially high level of diversity within this functional group. Moreover, AAP bacteria clearly co-vary with total cell abundance over different spatial and temporal scales, suggesting that the abundance of this group follows the same broad patterns as those for the total community. On the other hand, as much as there is variation within the group, evidence also suggests that AAP bacteria may be acting as a “guild” sharing features that may indeed set them apart from the rest of the bacterial community. For instance, AAP bacteria were consistently larger than the average bacterial cell in all lakes studied, and were positively related to DOC, and DOC:Chl*a*, whereas the total prokaryotic community did not follow these patterns. In addition, most AAP bacteria cells are within a size range that renders them extremely vulnerable to grazing [49,50], and it is very likely that AAP bacteria may be preferentially removed and thus subject to strong predation control and viral infection [4]. High potential growth [4,6], combined with a consistently low relative abundance in most samples would indeed suggest a strong top-down control of the AAP bacteria guild as a whole [4,16]. The fact that the phototrophic function of AAP bacteria may be related not only to higher than average cellular activity [6,26], but also to enhanced DOC uptake or degradation capacity [3,12,17], points to a key ecological role of this group, that needs to be further explored, especially in freshwaters.

## Supporting Information

S1 Fig The relationship between AAP bacterial abundance and bacteriochlorophyll *a* (BChl*a*) concentration. (TIF)

S1 Table Location and environmental characteristics for the lakes in the boreal (BOR) region. Averages of environmental variables are presented for the summer of 2008. ^a^Frequency of sampling. Secchi, secchi disk mean depth; DO, dissolved oxygen; DOC, dissolved organic carbon; TP, total phosphorous.
(PDF)

S2 Table Location and environmental characteristics for the lakes in the eastern townships (EST) region. Averages of environmental variables are presented for the summer of 2008**.** ^a^Frequency of sampling. Secchi, secchi disk mean depth; DO, dissolved oxygen; DOC, dissolved organic carbon; TP, total phosphorous.
(PDF)

S3 Table Location and environmental characteristics for the lakes in the laurentians (LAU) region. Averages of environmental variables are presented for the summer of 2008**.** ^a^Frequency of sampling. Secchi, secchi disk mean depth; DO, dissolved oxygen; DOC, dissolved organic carbon; TP, total phosphorous.
(PDF)

S4 Table Total bacterial abundance and the contribution of AAP bacteria to total bacterial abundance (%AAP) in the three broad seasonal categories: summer (stratification period), winter (ice cover period) and a mixed period. Average values are presented for each one of the 43 lakes sampled.
(PDF)
