# Supplementary figures and images for: Patterns in Abundance, Cell Size and Pigment Content of Aerobic Anoxygenic Phototrophic Bacteria along Environmental Gradients in Northern Lakes
Source: PLoS One. 2015 Apr 30;10(4):e0124035. doi: 10.1371/journal.pone.0124035 (PMC4415779; doi:10.1371/journal.pone.0124035)

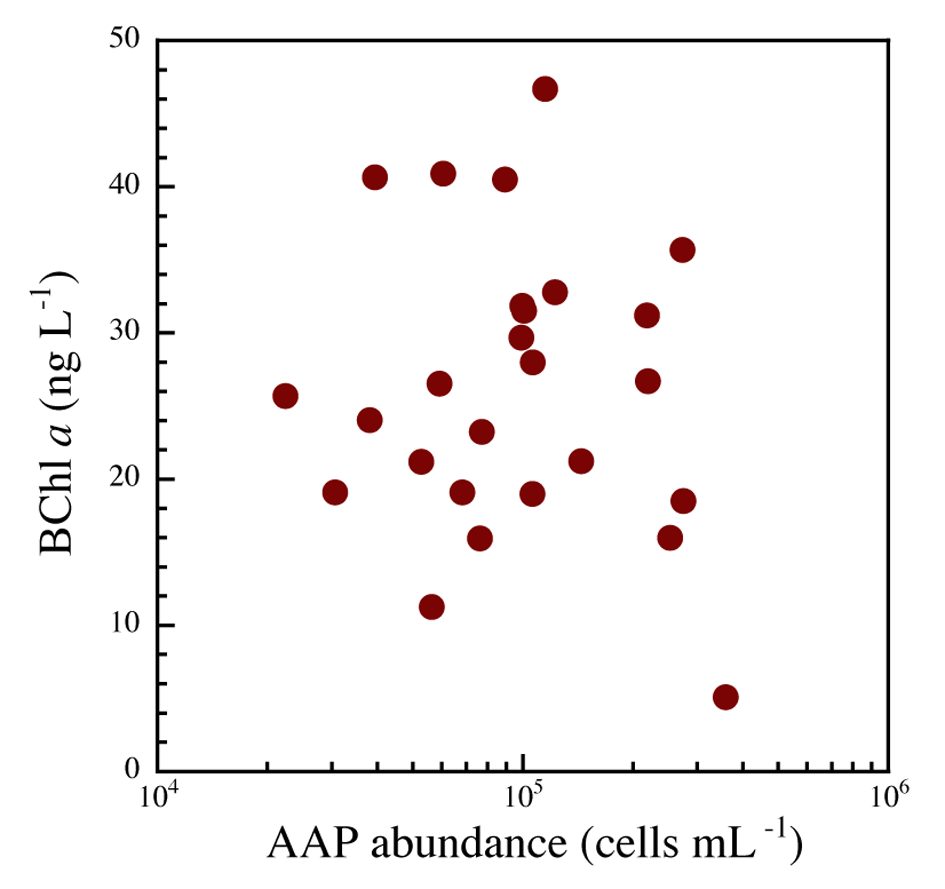

Supplement: S1 Fig — (TIF) [file pone.0124035.s001.tif]
