# Supplementary material for: Patterns in Abundance, Cell Size and Pigment Content of Aerobic Anoxygenic Phototrophic Bacteria along Environmental Gradients in Northern Lakes
Source: PLoS One. 2015 Apr 30;10(4):e0124035. doi: 10.1371/journal.pone.0124035 (PMC4415779; doi:10.1371/journal.pone.0124035)
Supplement: S1 Table — Averages of environmental variables are presented for the summer of 2008. aFrequency of sampling. Secchi, secchi disk mean depth; DO, dissolved oxygen; DOC, dissolved organic carbon; TP, total phosphorous. (PDF) [file pone.0124035.s002.pdf]

S1 Table. Location and environmental characteristics for the lakes in the boreal (BOR) region. Averages of environmental variables are presented for the summer of 2008**.** [a]Frequency of sampling. Secchi, secchi disk mean depth; DO, dissolved oxygen; DOC, dissolved organic carbon; TP, total phosphorous.

								Summer 2008 epilimnetic data					
Lake	**Region**	**Latitude & Longitude**	**Lake Area (km^2)**	**Water volume ($\times 10^3$ m^3)**	**Max. depth (m)**	**Secchi (m)**	**Freq[a]**	**Water temp (°C)**	**DO (%)**	**DO (mgL^{-1})**	**DOC (mgL^{-1})**	**TP (mgL^{-1})**	**Chl*a* (μgL^{-1})**
Brendon	BOR	52°3'N 75°30'W	1.07	3433	16.1	3.3	5	19.0	100	9.3	5.63	11.47	2.62
Clarkie	BOR	52°14'N 75°28W	24.69	616089	13.5	2.6	6	19.0	96.7	8.96	6.84	19.38	2.34
EM 320	BOR	52°9'N 76°7'W	0.48	846	5.6	2	4	19.3	97.1	8.96	8.96	9.3	2.37
L 12	BOR	52°17'N 75°26'W	nd	nd	5.7	3	1	16.3	91.2	8.92	7.81	7.13	2.90
L 40	BOR	52°1'N 75°31'W	0.16	662	10.3	3	6	19.0	99.9	9.26	5.15	8.56	2.34
L 48	BOR	52°10'N 75°36'W	nd	nd	7	2	1	17.2	94.9	9.14	6.83	6.27	2.41
L 56	BOR	52°10'N 75°44'W	nd	nd	5.8	2	1	18.1	88.7	8.37	4.23	11.50	3.40
L 9	BOR	52°11'N 75°44'W	nd	nd	3.8	2.3	1	17.7	91.0	8.67	9.33	8.12	2.80
L 11	BOR	52°9'N 75°45'W	0.38	485	5.3	1.6	5	20.3	98.6	8.93	10.30	13.2	1.59
L 2	BOR	52°7'N 75°49'W	0.043	51	1.2	1.3	4	18.0	99.8	9.45	9.49	12.37	2.43
L 34	BOR	51°58'N 75°46'W	0.46	1861	9.8	1.8	4	18.9	97.7	9.08	9.70	31.09	2.34
L 60	BOR	52°13'N 75°45'W	1.38	7282	13.5	2.75	4	19.0	100	9.22	5.93	4.40	1.84
L 66	BOR	51°57'N 76°0'W	0.072	267	10	1.8	3	18.6	97.6	9.13	8.86	12.72	2.05
L 8	BOR	52°7'N 75°43'W	0.32	703	9.7	2.4	4	19.3	99.8	9.1	9.50	8.01	2.43
Laby	BOR	52°13'N 75°42'W	2.57	3515	18	2.25	5	19.4	97.9	9.0	10.18	6.09	1.91
Mitsu	BOR	52°9'N 76°9'W	3.97	6858	8.6	2.6	4	18.2	98.2	9.26	7.16	12.17	1.95
Natel	BOR	52°10'N	3.87	17596	13.1	2.3	5	19.1	98.9	9.17	7.29	7.73	2.02

		75°40’W											
