# Supplementary material for: Patterns in Abundance, Cell Size and Pigment Content of Aerobic Anoxygenic Phototrophic Bacteria along Environmental Gradients in Northern Lakes
Source: PLoS One. 2015 Apr 30;10(4):e0124035. doi: 10.1371/journal.pone.0124035 (PMC4415779; doi:10.1371/journal.pone.0124035)
Supplement: S2 Table — Averages of environmental variables are presented for the summer of 2008. aFrequency of sampling. Secchi, secchi disk mean depth; DO, dissolved oxygen; DOC, dissolved organic carbon; TP, total phosphorous. (PDF) [file pone.0124035.s003.pdf]

S2 Table. Location and environmental characteristics for the lakes in the eastern townships (EST) region. Averages of environmental variables are presented for the summer of 2008**.** [a]Frequency of sampling. Secchi, secchi disk mean depth; DO, dissolved oxygen; DOC, dissolved organic carbon; TP, total phosphorous.

								Summer 2008 epilimnetic data					
Lake	**Region**	**Latitude & Longitude**	**Lake Area (km^2)**	**Water volume ($\times 10^3$ m^3)**	**Max. depth (m)**	**Secchi (m)**	**Freq[a]**	**Water temp (°C)**	**DO (%)**	**DO (mgL^{-1})**	**DOC (mgL^{-1})**	**TP (mgL^{-1})**	**Chl*a* (μgL^{-1})**
Argent	EST	45°18'N 72°19'W	2.91	4411	8.5	2.4	1	20.6	88.6	7.97	10.41	14.75	1.83
Bowker	EST	45°24'N 72°14'W	2.48	60499	36.5	8	16	21.7	101	8.94	2.31	3.56	0.79
Brompton	EST	45°28'N 72°7'W	22.63	113211	25	2.3	1	22.1	104	9.13	6.03	10.37	4.99
Bran de Scie	EST	45°24'N 72°12'W	nd	nd	8.5	1.9	13	23.1	105	9.03	6.44	16.43	11.66
Brome	EST	45°16'N 72°30'W	15.60	78150	10.09	2	1	21.5	105	9.32	9.48	22.22	7.43
Fraser	EST	45°22'N 72°10'W	9.34	13390	11.9	2.2	1	19.9	91.9	8.37	5.51	7.86	2.75
Orford	EST	45°17'N 72°16'W	1.32	21467	31.5	4.4	1	21.1	102	9.14	3.68	4.26	1.67
Parker	EST	45°20'N 72°18'W	nd	nd	8.5	1.4	1	20.0	89.8	8.17	12.78	26.23	3.50
Roxton	EST	45°28'N 72°39'W	0.42	nd	4.8	1.5	1	21.9	93.2	8.16	12.11	40.31	14.24
Simoneau	EST	45°24'N 72°11'W	nd	nd	22.2	3	1	20.7	83.1	7.48	5.21	5.03	1.77
Stuckley	EST	45°22'N 72°15'W	1.03	50789	23.5	3.5	1	20.1	92.7	8.42	7.07	6.38	3.54
Tomcod	EST	45°3'N 72°2'W	0.83	1355	2.7	0.25	1	19.1	47.6	4.37	10.90	177.20	55.34
Waterloo	EST	45°21'N 72°30'W	1.61	3766	4.5	0.9	1	20.5	105	9.75	12.22	38.06	20.04
