# Supplementary material for: Patterns in Abundance, Cell Size and Pigment Content of Aerobic Anoxygenic Phototrophic Bacteria along Environmental Gradients in Northern Lakes
Source: PLoS One. 2015 Apr 30;10(4):e0124035. doi: 10.1371/journal.pone.0124035 (PMC4415779; doi:10.1371/journal.pone.0124035)
Supplement: S3 Table — Averages of environmental variables are presented for the summer of 2008. aFrequency of sampling. Secchi, secchi disk mean depth; DO, dissolved oxygen; DOC, dissolved organic carbon; TP, total phosphorous. (PDF) [file pone.0124035.s004.pdf]

S3 Table. Location and environmental characteristics for the lakes in the laurentians (LAU) region. Averages of environmental variables are presented for the summer of 2008**.** [a]Frequency of sampling. Secchi, secchi disk mean depth; DO, dissolved oxygen; DOC, dissolved organic carbon; TP, total phosphorous.

								Summer 2008 epilimnetic data					
Lake	**Region**	**Latitude & Longitude**	**Lake Area (km^2)**	**Water volume ($\times 10^3$ m^3)**	**Max. depth (m)**	**Secchi (m)**	**Freq[a]**	**Water temp (°C)**	**DO (%)**	**DO (mgL^{-1})**	**DOC (mgL^{-1})**	**TP (mgL^{-1})**	**Chl*a* (μgL^{-1})**
Connelly	LAU	45°53'N 73°57'W	1.25	9211	23	3.5	13	23.0	117	10.48	4.34	10.20	3.73
Cornu	LAU	45°58'N 74°23'W	nd	nd	13.1	4.1	1	23.6	123	10.51	3.74	6.94	1.11
Croche	LAU	45°59'N 74°0'W	0.18	838	9.8	4	12	22.8	98.7	8.39	4.45	5.59	3.21
Cryistal	LAU	45°58'N 73°53'W	0.29	2820	19.2	7.1	1	22.6	99.4	8.6	2.60	4.53	1.21
Duffy	LAU	46°09'N 73°55'W	nd	nd	1.1	1.1	1	24.9	92.0	7.75	7.45	10.55	2.65
Dupuis	LAU	46°4'N 73°52'W	nd	nd	45.4	3.3	1	22.4	101	8.83	5.17	6.09	2.28
Echo	LAU	45°53'N 74°1'W	nd	nd	2.1	2.1	1	25.0	126	10.45	5.26	15.29	3.54
En Coeur	LAU	45°58'N 74°0'W	0.43	1284	8	3.6	1	21.9	105	9.25	4.58	8.57	4.62
Fournelle	LAU	45°54'N 74°2'W	0.18	485	7.2	4.3	1	23.5	118	10.06	4.02	9.13	1.42
Lac du Nord	LAU	45°48'N 74°7'W	nd	nd	7.8	2.75	1	21.1	105	9.33	6.13	31.16	4.19
Morency	LAU	45°55'N 74°2'W	0.25	2223	8.5	4.85	1	22.6	114	9.92	3.45	7.65	1.89
Pin Rouge	LAU	45°57'N 74°2'W	0.13	670	6.9	1.9	1	21.6	113	9.96	7.96	12.60	2.49
Rond	LAU	45°58'N 74°2'W	0.094	428	10.5	3	1	23.5	92.8	7.85	4.79	10.69	2.81
