# Supplementary material for: Patterns in Abundance, Cell Size and Pigment Content of Aerobic Anoxygenic Phototrophic Bacteria along Environmental Gradients in Northern Lakes
Source: PLoS One. 2015 Apr 30;10(4):e0124035. doi: 10.1371/journal.pone.0124035 (PMC4415779; doi:10.1371/journal.pone.0124035)
Supplement: S4 Table — Average values are presented for each one of the 43 lakes sampled. (PDF) [file pone.0124035.s005.pdf]

S4 Table. Total bacterial abundance and the contribution of AAP bacteria to total bacterial abundance (%AAP) in the three broad seasonal categories: summer (stratification period), winter (ice cover period) and a mixed period. Average values are presented for each one of the 43 lakes sampled.

Lake	Region	Summer		Winter		Mix	
		AAP (%)	Total bacteria (×10^5 cellmL^{-1})	AAP (%)	Total bacteria (×10^5 cellmL^{-1})	AAP (%)	Total bacteria (×10^5 cellmL^{-1})
**Brendon**	BOR	5.5	34.3	4.38	42.4	-	
**Clarky**	BOR	5.0	13.9	3.57	29.9	9.38	20.4
**EM 320**	BOR	8.9	21.5	-	-	-	-
**L 12**	BOR	9.0	17.9	-	-	-	-
**L 40**	BOR	7.7	26.2	2.17	73.3	-	-
**L 48**	BOR	3.9	6.05	-	-	-	-
**L 56**	BOR	7.8	12.2	-	-	-	-
**L 9**	BOR	5.7	9.81	-	-	-	-
**L 11**	BOR	11.5	39.1	4.12	38.6	-	-
**L 2**	BOR	9.5	37.3	1.62	38.9	4.00	16.0
**L 34**	BOR	6.0	26.2	-	-	-	-
**L 60**	BOR	6.0	17.8	3.91	26.0	-	-
**L 66**	BOR	11.3	17.8	-	-	-	-
**L 8**	BOR	6.1	31.7	-	-	-	-
**Laby**	BOR	7.9	14.3	1.48	27.6	-	-
**Mitsu**	BOR	9.8	34.1	1.67	73.7	-	-
**Natel**	BOR	7.1	15.2	3.2	48.0	-	-
**Argent**	EST	16.1	20.0	-	-	-	-
**Bowker**	EST	4.9	13.2	3.64	26.3	3.49	20.1
**Brompton**	EST	6.6	28.3	-	-	-	-
**Bran de Scie**	EST	9.7	26.1	2.00	39.4	1.94	28.2
**Brome**	EST	5.3	10.7	-	-	-	-
**Fraser**	EST	4.5	32.0	-	-	-	-
**Orford**	EST	4.1	14.6	-	-	-	-
**Parker**	EST	8.6	12.4	-	-	-	-
**Roxton**	EST	7.4	29.7	-	-	-	-
**Simoneau**	EST	5.8	19.7	-	-	-	-
**Stuckley**	EST	3.2	3.80	-	-	-	-
**Tomcod**	EST	8.3	66.4	-	-	-	-
**Waterloo**	EST	2.2	47.3	-	-	-	-
**Connelly**	LAU	2.1	32.7	0.06	19.1	10.0	27.2
**Cornu**	LAU	3.3	25.0	-	-	-	-
**Croche**	LAU	1.8	16.0	4.43	11.9	8.66	18.2
**Cryistal**	LAU	4.6	14.8	-	-	-	-
**Duffy**	LAU	1.8	31.5	-	-	-	-
**Dupuis**	LAU	6.1	24.3	-	-	-	-
**Echo**	LAU	8.3	43.9	-	-	-	-
**En Coeur**	LAU	5.0	21.3	-	-	-	-
**Fournelle**	LAU	4.8	17.5	-	-	-	-
**Lac du Nord**		3.5	19.1	-	-	-	-
**Morency**	LAU	6.0	36.5	-	-	-	-
**Pin Rouge**	LAU	7.1	25.2	-	-	-	-
**Rond**	LAU	3.3	25.1	-	-	-	-
